# Community-Based Interventions to Improve Eye Health Outcomes in Older Adults: A Systematic Review and Meta-Analysis

**DOI:** 10.3389/phrs.2025.1607404

**Published:** 2026-01-21

**Authors:** Wasana Luangphituck, Plernpit Boonyamalik, Piyanee Klainin-Yobas, Sunee Lagampan, Chukiat Viwatwongkasem

**Affiliations:** 1 Department of Public Health Nursing, Faculty of Public Health, Mahidol University, Thailand; 2 Department of Public Health Nursing, Faculty of Public Health, Mahidol University, Thailand; 3 The Alice Lee Center for Nursing Studies, Yong Loo Lin School of Medicine, National University of Singapore, Singapore; 4 Department of Biostatistics, Faculty of Public Health, Mahidol University, Thailand

**Keywords:** blindness, eye health, community-based intervention, community-dwelling older adults, systematic review

## Abstract

**Objectives:**

This review aimed to synthesize evidence on community-based interventions designed to improve eye health among older adults.

**Methods:**

Eleven electronic databases and reference lists of relevant studies were systematically searched. Two reviewers independently screened records, extracted data, and assessed study quality. Pooled effect sizes were calculated using a random-effects model with standardized mean differences (SMD) and relative risks. Heterogeneity was assessed using the I^2^ and Chi-square tests, with subgroup, sensitivity, and publication bias analyses performed.

**Results:**

Twenty-two studies met the inclusion criteria, and 13 were included in the meta-analysis. Interventions included educational, telephone-based, and health promotion programs. Educational programs significantly improved attitudes toward eye health (SMD = 3.91) and general eye health behaviors (SMD = 8.20). Structured teaching interventions had the greatest effect on knowledge (SMD = 4.04), while community-based support groups improved eye examination uptake (SMD = 4.33). Subgroup and meta-regression analyses found no significant moderators, with persistent heterogeneity.

**Conclusion:**

Community-based interventions appear to enhance eye health knowledge and behaviors among older adults, but evidence remains limited and heterogeneous, warranting cautious interpretation.

**Systematic Review Registration:**

PROSPERO: Identifier CRD42023434652.

## Introduction

Currently, over 2.2 billion people worldwide have a vision impairment, with at least one billion experiencing a preventable form [1]. The consequences of visual impairment extend beyond individual health, impacting families and societies through heightened social isolation, increased dependency ratios, a surge in demand for eye care services, and escalated government spending on healthcare costs [2].

Promoting eye health through effective programs is crucial for preventing visual impairment in older persons. A community-based approach, considered a gold standard for health promotion and disease prevention, offers several advantages, including accessibility to all risk groups, and the ability to influence contextual lifestyle factors. Studies on community-based interventions such as educational programs, behavioral modifications, and community health screenings have shown promise in improving health behavior, service access, literacy, and overall health outcomes. However, despite significant results reported in various studies [3–5], there is often a lack of high-quality data supporting the development of eye health promotion programs.

In addition, the best way to prevent blindness is through early detection of age-related eye diseases. There are various factors that could ensure early detection to prevent blindness. Promoting knowledge, attitudes, and practices regarding eye health is essential. Awareness and knowledge of eye diseases are important factors in prevention of various eye problems. Spreading knowledge will motivate people to visit an ophthalmologist to have an annual eye examination as a dilated fundus which is important in preventing age-related blindness [4].

Visual acuity is the performance of the visual system [6]. Visual acuity is also a crucial component in screening for eye diseases such as glaucoma, especially when symptoms may not manifest in the early stages. Assessing visual acuity helps detect eye problems early and aids in preventing or treating eye diseases appropriately before they cause significant damage or severe symptoms.

Behavioral and lifestyle factors can significantly contribute to the development and progression of eye conditions that may lead to vision impairment [7]. Eye health behavior is any activity undertaken by an individual who believes themselves to be healthy to prevent or identify eye diseases and vision impairment in an asymptomatic state. Encouraging the adoption of eye health-promoting activities and discouraging eye-harming behaviors are pivotal in preventing blindness [8].

Vision-related quality of life refers to the impact that visual health and functioning have on an individual’s overall quality of life. The concept encompasses both the physical and psychosocial aspects of vision, including how visual impairments or changes affect a person’s independence, emotional wellbeing, and social interactions. Existing evidence suggests that reduced visual acuity and visual field loss are both associated with worsening in vision-related quality of life [9].

Currently, it is uncertain the best way to develop interventions that will promote good eye health in community-dwelling older persons who are at high risk of blindness. Prior systematic reviews have demonstrated how effective community-based interventions are especially at identifying participants with diabetic retinopathy [10]. Additionally, ongoing and proposed reviews have explored various aspects of eyecare services outcomes [11]. The evidence for the best intervention for precisely enhancing various eye health outcomes in older people living in communities is seriously insufficient. Extensive searches in the Cochrane Database of Systematic Reviews, the JBI Database of Systematic Reviews and Implementation Reports, and the PROSPERO registration found no current systematic reviews on this crucial issue.

Therefore, this review aims to comprehensively synthesize the best state-of-the-art of community-based interventions on attitudes towards eye health, eye health knowledge, eye examination rate, eye health behavior, and vision-targeted health-related quality of life among non-visually impaired older people in community settings. The overarching goal is to provide supporting evidence for designing effective interventions aimed at improving eye health to prevent visual impairment among older people residing in communities.

### Review Question

Among older persons living in diverse community settings (P), do community-based interventions targeting eye health (I), compared to usual care or no intervention (C), improve eye health attitude, eye health knowledge, eye examination rate, visual acuity, eye health behavior, and vision-related quality of life (O), as evaluated in randomized controlled trials (RCTs), controlled clinical trials (CCTs), or quasi-experimental studies (S)?

## Methods

### Protocol Registration

A summary of the protocol and supplemental data for this systematic review has been registered on the International Prospective Register of Systematic Reviews (PROSPERO) under the registration number CRD42023434652.

### Eligibility Criteria

#### Participants

Participants were older persons residing in community, health settings, geriatric facilities, home-visit nursing, patients’ homes, and clinics (excluding hospital inpatients). The study excluded individuals with pre-existing age-related eye diseases.

#### Intervention

All type of community-based intervention: community as setting, community as target, community as agent and community as resource aimed at influencing change at least one of the three levels 1) personal level, such as health education and training 2) interpersonal level, such as establishing new social connections 3) at the community level, conducted by various health professionals, would be considered [12]. This study excluded interventions targeting older persons with specific eye diseases (e.g., cataracts, glaucoma), as well as vision rehabilitation, pre/post-surgery, and treatment programs, because these interventions focus on disease management or clinical care rather than prevention and health promotion.

#### Comparator

This review considered studies that compare no intervention control, active control, placebo control, and usual care.

#### Outcomes

Primary outcomes of the study included eye health attitude, eye health knowledge, eye examination rate, visual acuity, eye health behavior, and vision-related quality of life. Excluded are studies espicially focused on cost-effectiveness and interventions targeting specific eye diseases older persons.

### Type of Studies

This review included studies with experimental and clinical trial designs to ensure rigorous evaluation of intervention effectiveness. Eligible designs encompassed randomized controlled trials, cluster randomized trials, and quasi-experimental studies. No restrictions were placed on the year of publication, allowing for a comprehensive inclusion of both older and more recent evidence. Only studies published in English or Thai were considered, as these were the languages accessible to the review team.

### Information Sources

PubMed, MEDLINE, CINAHL, ScienceDirect, Ovid, and Scopus were systematically searched for published studies. Grey literature sources included Google Scholar, ProQuest Dissertations and Theses Global, Ethos, Thai Digital Collection, and Thai Journal Online (ThaiJO). A manual search was conducted in major journals such as JAMA Ophthalmology, the American Journal of Ophthalmology, Ophthalmology Update, and the Thai Journal of Ophthalmology. Reference lists of identified sources were manually searched, and major journals underwent a hand search.

### Search Strategies

The search strategy, guided by the PRESS checklist [13], covered both published and unpublished research. Boolean and proximity operators were used to combine search terms. A preliminary search of PubMed was conducted to locate articles on this topic, which was initially covered (Supplementary Appendices SA). Comprehensive search approaches were tailored for each database, incorporating keywords, index terms, and consultation with a librarian. A reference list of all listed sources of evidence was screened for further study.

### Study Selection

Keywords and Medical Subject Headings (MeSH) terms (e.g., *“eye OR vision OR ocular OR visual OR ophthalmic AND program OR intervention OR training OR education AND aged OR elderly OR senior OR older people OR geriatric”*) were tested in PubMed. Following the search, all citations found were gathered and uploaded into Endnote version 9 [14], with duplicates deleted. Two independent reviewers assessed titles, abstracts, and full texts against inclusion criteria. Disagreements were resolved through discussion or with the assistance of a third reviewer. The Preferred Reporting Items for Systematic Reviews and Meta-Analyses (PRISMA) flow diagram, which summarizes the study selection process, was used in this study [15].

### Assessment of Methodological Quality

A pilot test of six studies was conducted to ensure consistency between the two reviewers in study selection and data extraction. Prior to inclusion in the review, two independent reviewers assessed the methodological quality of each eligible study using the Cochrane Risk of Bias tool for randomized trials (RoB1) [16] and standardized critical appraisal instruments from the Joanna Briggs Institute Meta-Analysis of Statistics Assessment and Review Instrument (JBI-MAStARI). Selection, performance, detection, attrition, and reporting biases were assessed, with each domain rated as yes, no, or uncertain. All studies were included regardless of risk-of-bias scores, and study authors were contacted for clarification when needed. The GRADE approach was used to evaluate the overall quality of evidence, grading each outcome as high, moderate, low, or very low based on study design, risk of bias, imprecision, inconsistency, indirectness, and publication bias. Reviewer discrepancies were resolved through discussion or consultation with additional reviewers.

### Data Extraction

Two independent reviewers extracted data from the studies included in the review. Specific information on demographics, interventions, comparisons, results, findings, and relevance to the review’s purpose was collected. Discrepancies were resolved through discussion or with the input of a third reviewer. Authors were contacted for missing or additional details when necessary.

### Data Synthesis and Presentation

Meta-analyses were performed using the Review Manager Software [17]. Effect size was the primary measure of association, enabling standardized comparisons across studies. Standardized mean differences (SMD) with 95% confidence intervals (CI) were calculated for continuous outcomes, and risk ratios (RR) for dichotomous outcomes. Statistical significance was defined as *p* < 0.05. Heterogeneity was assessed using Chi-square and I^2^, with I^2^ > 50% indicating substantial heterogeneity. Subgroup analyses and meta-regression were performed as appropriate. When outcome measures varied between studies, a narrative summary was provided. When ≥10 studies were available, funnel plots were used to assess publication bias.

## Results

### Search Results

**FIGURE 1:**
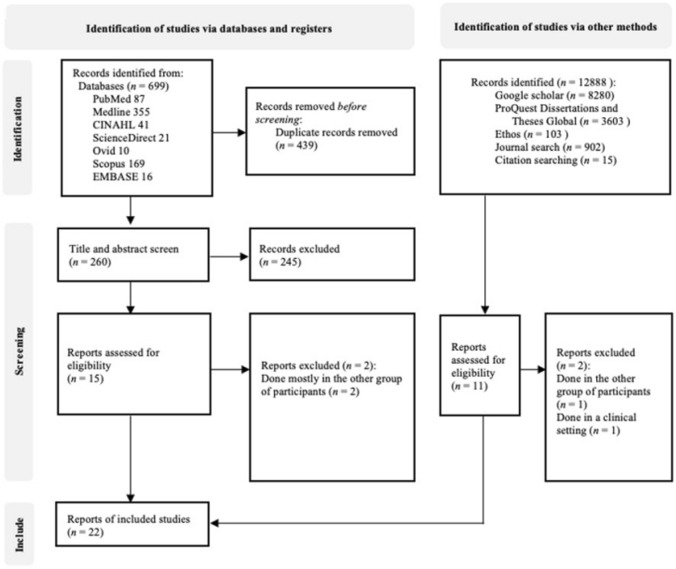
The Preferred Reporting Items for Systematic Reviews and Meta-Analyses diagram of the search results (Thailand, 2025).

Studies were initially identified through database searches and additional sources, yielding 699 records from electronic databases, of which 439 duplicates were removed, and an additional 12,888 records from other sources. The remaining 260 studies were screened based on titles and abstracts, and 245 were excluded because they did not meet the predefined inclusion criteria, such as not focusing on community-based eye health interventions, involving participants outside the target age group, or lacking relevant outcomes. Full-text articles were then retrieved for detailed assessment—15 from databases and 11 from other sources—and four were excluded because they were conducted primarily in other population groups or in clinical settings. This process resulted in 22 studies being included in the final synthesis (Figure 1).

### Study Characteristics

There were 13 randomized controlled trials, five controlled clinical trials, and four quasi-experimental trials included in this systematic review. At baseline, sample sizes ranged from 50 to 1,695. The trials were reported in English (n = 20) and Thai (n = 2) between 1999 and 2022. Included studies were conducted in eight different countries: Australia, Canada, India, Iran, Kenya, Thailand, Vietnam, and the United States of America.

The community-based intervention was tested on different outcomes: eye examination rate (n = 15), eye health knowledge (n = 7), eye health attitude (n = 4), and eye health behavior (n = 3). Other outcomes consisted of various eye health behavioral aspects such as visual acuity, wearing sunglasses, diabetic retinopathy practice, perceived self-efficacy related to eye health promotion behavior, and vision-related quality of life (n = 1). Data were obtained at different assessment points: post-intervention, 1–3 months post-intervention, 6 months post-intervention, 12 months post-intervention, and 3 years follow-up. The characteristics of the included studies are summarized in Table 1.

**TABLE 1:** Characteristics of the included studies (Thailand, 2025).

No.	Authors	Study design	Participants	Age range (mean age), year	Sex, %	Sample size	Intervention	Comparator	Outcomes measured
1	Basch et al. [18]	RCT	1. Diagnosis of diabetes mellitus 2. African American ethnicity 3. Age 18 years or older 4. No documentation of a dilated retinal examination in the preceding 14 months	(55.6) SD = 12.9	F, 65.7 M, 34.3	IG = 130 CG = 143	The multicomponent educational intervention	Usual care, Mailing of a meal-planning booklet	Dilated retinal examination rate
2	Anderson et al. [19]	RCT	Diabetic patients residing in the community and attending eye screening clinics at eight different locations	20–65, >65 (55)	F, 70.1 M, 29.9	IG = 67 CG = 65	Personalized follow-up interventions	Usual care	Diabetes eye evaluation
3	Conlin et al. [20]	RCT	Participants with diabetes mellitus	(67)	F, 78.1 M, 21.9	IG = 223 CG = 225	Tele retinal imaging program	Usual care	Dilated eye examinations
4	Walker et al. [21]	RCT	1. Aged 18 years or older 2. Diagnosed with diabetes mellitus 3. Have access to a telephone 4. Report no dilated fundus examination (DFE) in the previous 12 months	(56.6) SD = 12.5	F, 60 M, 40	IG = 305 CG = 293	Tailored telephone intervention	A mailed print intervention	Documentation of a dilate fundus examination (DFE)
5	Hazavehei et al. [22]	RCT	1. Non–insulin-dependent diabetes, age 40–60, diabetes duration >5 years 2. At risk of ocular complications	40–60 (54.40)	F, 72 M, 22	IG = 50 CG = 50	Educational program based on the BASNEF model (belief, attitude, subjective norm, and enabling factors)	Usual care	1. Patients’ knowledge 2. BASNEF components (enabling factors, attitude, subjective norm, normative norms), intention towards behaviors, and patient behavior
6	Ellish et al. [23]	RCT	1. Community-dwelling African americans aged ≥65 years 2. No dilated fundus examination (DFE) in the past 2 years	65–80, >80	F, 73.8 M, 26.2	IG = 164 CG = 165	A tailored individualized intervention	Targeted print intervention	Eye doctor–confirmed dilated fundus examinations (DFE)
7	Owsley et al. [24]	RCT	African American communities and age >60 years	(75)	F, 87 M, 13	IG = 54 CG = 63	The eye health education program “InCHARGE”	The social contact control arm	Eye care utilization, attitudes and belief about eye care
8	Chew et al. [25]	RCT	1. At risk for developing choroidal neovascularization (CNV) 2. Best-corrected visual acuity of 20/60 or better in the study eye(s) 3. No media opacities preventing quality fundus photography and no other retinal disorders, such as diabetic retinopathy	(72.5)	F, 59 M, 41	IG = 51 CG = 30	Home monitoring with Foresee home device using macular visual field testing with hyperacuity techniques and tele-monitoring	Standard care	Best-corrected visual acuity scores
9	Weiss et al. [26]	RCT	1. African American individuals 65 years and older 2. Confirmed diagnosis diabetes mellitus 3. Had not obtained a dilate fundus examination in the preceding 12 months	(72.8)	F, 68 M, 51	IG = 103 CG = 103	Behavioral activation for diabetic retinopathy prevention intervention	Placebo treatment (supportive therapy)	1. Dilate fundus examination 2. Risk perceptions and risk knowledge of diabetes mellitus and complication such as diabetic retinopathy 3. Vision targeted health-related quality of life (NEI-VFQ 25)
10	Crossland et al. [27]	RCT	1. 18 years of age or older 2. Confirmed diagnosis of type 2 diabetes mellitus	(68.3)	F, 49 M, 51	IG = 174 CG = 181	General practice-based diabetic retinopathy screening via annual cycle of care	Usual care	Timely and appropriate DR screening
11	Zangalli et al. [28]	RCT	1. 18 years of age or older 2. Had no, mild, or moderate DR 3. Had been recommended for a follow-up DFE but had not scheduled a subsequent visit	19–95 (61)	F, 68.3 M, 31.7	IG = 262 CG = 259	A multipronged intervention: Education-and telephone-based intervention	Usual care	Diabetic dilated fundus examination (DFE)
12	Mwangi et al. [3]	RCT	1. 18 years of age or older 2. Members of a diabetes support group 3. Had not undergone a screening exam in the past 12 months	(66.4)	F, 41 M, 59	IG = 51 CG = 54	Community-based diabetic support groups (DSGs) peer–led health education intervention	Usual care	Rates of uptake eye examination
13	Paudel et al. [29]	RCT	1. Aged ≥30 years 2. Resided in the household for at least 6 months each year 3. Take meal from the same kitchen	30–90 (51.5)	F, 65.7 M, 34.3	IG = 200 CG = 200	A community-based eye health education intervention	Usual care	1. Awareness and knowledge of eye health 2. Use of eye care services 3. Reasons for not wearing eyeglasses
14	Na nakorn et al. [30]	CCT	1. Aged ≥60 years 2. Members of the senior citizens club	60–69	F, 75 M, 25	IG = 20 CG = 20	A perceived benefit promotion program	Usual care	Eye health promoting behavior
15	Boonruan et al. [31]	CCT	1. Aged ≥60 years 2. Visual acuity better than 20/70 3. No history of eye disease or eye surgery 4. Members of the senior citizens club	(68)	-	IG = 40 CG = 40	A health education program	Usual care	1. Perceived benefits of performing eye health protecting behaviors 2. Perceived self-efficacy to perform eye health promotion behaviors 3. Eye health promotion behaviors
16	Sapru et al., USA, 2017	CCT	1. African americans ≥50 years and all adults ≥60 years 2. African American and hispanics ≥40 years, whites ≥50 years 3. Any age or race with diabetes mellitus, glaucoma associated diagnosis, or family history of glaucoma	40–60, ≥60	-	IG = 707 CG = 518	A mobile community-based eye health education program	A telemedicine program	1. A new glaucoma related case 2. Glaucoma knowledge
17	Panchal and batra [4]	CCT	1. Aged 50–70 years 2. Participants diagnosed with or receiving treatment for any visual impairment were excluded	50–70	F, 54 M, 46	IG = 50 CG = 50	Structured teaching programme (STP)	Usual care	1. Knowledge 2. Attitude
18	Ramagiri et al. [5]	CCT	1. People with diabetes mellitus who taking oral hypoglycemic agent or insulin on prescription from an endocrinologist 2. Had not yet visited an ophthalmologist	7–80 (53)	F, 56.9 M, 43.1	IG = 131 CG = 104	Health education intervention with watched videos	Health education intervention with pamphlet	Uptake of diabetic retinopathy screening
19	Müller et al., Australia, 2007	CCT	1. Aged 70–79 years with diabetes 2. Participants with a family history of glaucoma or age-related macular degeneration who reported changes in vision	74.5 (70–79)	F, 55.5 M, 45.5	IG = 1,695,1728	Eye health promotion campaign	-	Utilization of eye care services
20	Hark et al. [28]	Quasi-experimental	African americans aged over 50 and adults aged over 60 living in underserved communities in Philadelphia	​	​	IG = 1,508	Community-based intervention to improve detection and management of glaucoma	-	Eye examination (glaucoma detection examination)
21	Umaefulam et al. [32]	Quasi-experimental	1. Women aged >18 years 2. Individuals with diabetes, at risk for diabetes mellitus, or with a family history of gestational diabetes	18–69	-	IG = 50	Mobile health intervention	-	1. Diabetic knowledge 2. Diabetic attitude 3. Diabetic practice
22	Rhodes et al., USA, 2022	Quasi-experimental	1. African americans ≥40 years 2. White ≥50 years 3. Diabetes 4. Family history of glaucoma	20–90 (55.1)	F, 65.3 M, 34.7	IG = 518	Eye health education intervention	-	1. Knowledge about glaucoma 2. Attitudes about eye care

RCT: randomized control trial; CCT: clinical control trial; IG: intervention group; CG: comparator group; F: female; M: male; SD: standard deviation.

### Intervention Characteristics

Interventions were provided in different settings including home, communities, and primary care settings. The intervention contents included an educational program, tailored telephone intervention, tele-retinal screening program, telephone call, behavioral activation program, community-based support group and letter reminder. The intervention characteristics of the included studies are summarized in Table 2.

**TABLE 2:** Description of community-based intervention (Thailand, 2025).

No.	Author Country, Year	Intervention content	Frequency/Duration	Number of sessions	Mode of delivery/provider	Measurement point	Main results
1	Basch et al. [18]	The multicomponent educational intervention • Nine-page color booklet covering two main topics: What diabetic retinopathy is and how to manage it • Motivational videotape designed to encourage annual dilated retinal examinations and promote booklet use • Individually tailored tip-sheet mailings providing practical strategies to overcome specific barriers • Semi-structured telephone education delivering one-on-one interactive counseling and education	4 telephone calls and 53 min per person	4 main activities	Individual, group/Health educator	Within 6 months after randomization	The odds ratio for receiving a retinal examination associated with the intervention was 4.3 (95% CI = 2.4, 7.8). The examination rate pooled across sites was 54.7% (n = 73/130) in the intervention group and 27.3% (n = 39/143) in the control group
2	Anderson et al. [19]	Personalized follow-up interventions • Invitation letter providing the date, time, and location of the upcoming eye clinic and encouraging patients to call a toll-free number to schedule an appointment • Follow-up phone call made to patients who had not scheduled a diabetes eye examination (DEE) within 10 days of the letter being sent	​	2 main activities	Individual/Volunteer community-based ophthalmologist	Pre-intervention, 12 months after intervention	The return rate for the intensive, personalized follow-up group was 66%, significantly (*p* = 0.001) higher than the 35% return rate for the standard follow-up group
3	Conlin et al. [20]	Tele retinal imaging program • Imaging protocol: Single-frame video images of three 45° retinal fields were captured using a nonmydriatic retinal camera interfaced with a standard color video camera • Education component: Educated on the importance of optimal blood glucose and blood pressure control the basic anatomy of the ocular fundus was demonstrated, highlighting the optic nerve, macula, and retinal blood vessels, and guidance was provided to establish an appropriate eye-examination schedule	​	2 main parts	Individual/Trained imagers	Pre-intervention, Within 12 months after intervention	During the 12 months following the randomization visit, participants who received teleretinal imaging (*n* = 223) were more adherent to follow-up dilated eye exams by an eye care professional than those who did not have imaging (*n* = 225) (87% vs. 77%, *p* < 0.01)
4	Walker et al. [21]	Tailored telephone intervention • Assessment of stage of change: Evaluating readiness to undergo a dilated fundus examination (DFE) • Problem-solving skills: Teaching basic strategies to overcome personal, motivational, and institutional barriers preventing participants from obtaining a DFE • Diabetes self-management education: Tailored telephone calls to motivate about the importance of annual dilated eye exams, identify barriers, and communicate associated risks	7 telephone calls per person/6 months	3 main activities	Individual/Bilingual interventionist	Within 6 months after randomization	There was a 74% increase in retinopathy screening in the telephone (n = 103/305) versus print group (n = 57/293) (*p* < 0.0005, RR = 1.74, 95% CI = 1.31–2.30, χ^2^ = 15.63)
5	Hazavehei et al. [22]	Educational program based on the BASNEF model (belief, attitude, subjective norm, and enabling factors) • Diabetes education: Overview of diabetes, its effects on the eyes, and potential ocular complications • Nutrition and medication: Impact of proper diet and adherence to medication in preventing ocular complications • Educational participation: Emphasis on attending educational sessions • Physical activity: Role of regular exercise in controlling blood sugar • Ophthalmology visits: Importance of regular eye examinations • Family involvement: Patients’ families participated in one of the educational sessions	55–60 min per sessions/1 month	6 educational sessions	Group of participants/Ophthalmologist, specialist in diabetes, nutrition experts	Pre-intervention, Post-intervention, 3 months after intervention	Immediately and 3 months post-intervention, the experimental group showed significantly higher scores than the control group in knowledge, behavioral outcomes, attitudes, enabling factors, normative beliefs, subjective norms, intentions, and behavior (*p* < 0.001)
6	Ellish et al. [23]	The tailored, individualized intervention was developed based on the health belief model, Transtheoretical model, and Precaution adoption process model, which guided the design and implementation of the program • Four-page newsletter: Comprised six sections, including a testimonial to model eye examination behavior and a barrier table providing strategies to overcome specific obstacles • Tailored content: Each newsletter contained individualized messages based on participants’ responses to selected baseline questionnaire items • Follow-up telephone calls: Conducted at 1, 3, and 6 months to reinforce content and provide additional support	/6 months	2 sessions	Group of participants	Pre-intervention, 6 months after intervention	No significant difference was noted in this measure by intervention group (RR, 1.07; 95% CI, 0.82–1.40), with 66 participants in the tailored group (40.2%) and 62 participants in the targeted group (37.6%) having an eye doctor–confirmed dilated fundus examinations (DFE
7	Owsley et al. [24]	The eye health education program “InCHARGE” • Booklet based on theories of health behavior, including the empowerment model, health belief model, and social learning Theory, was developed with three main sections: 1) Being InCHARGE of your eye health: Understanding prevention, common eye problems, and the components of a comprehensive eye exam 2) Being InCHARGE of solving common challenges: Strategies for finding an eye doctor, arranging transportation, covering exam costs, and communicating effectively with healthcare providers 3) Being InCHARGE of eye care: Setting goals to undergo an annual dilated comprehensive eye exam and making a personal commitment to eye health	​	3 main parts of curriculum	Group of participants/Trained instructor	Pre-intervention, 12 months after intervention	There were no group differences 6 months post-event. For 12 months pre-event, dilated exam rate was similar in the groups (38.3% InCHARGE, 40.8% control), and unchanged during 12 months post-even
8	Chew et al. [25]	A home device using macular hyperacuity testing with tele-monitoring for remote visual tracking • Participants received a home monitoring device with instructions for installation and use • Encouraged to use the device on a daily basis and results were transmitted automatically via cellular modem to a central data monitoring center • Any change from baseline triggered an alert to the clinical team, prompting an ophthalmologist visit within 72 h	​	​	Individual/Certified examiners	Pre-intervention, at choroidal neovascularization (CNV) detection	The device arm showed a smaller decline in visual acuity (median −4 letters, IQR −11 to −1) than standard care (median −9 letters, IQR −14 to −4; *p* = 0.021)
9	Weiss et al. [26]	Behavioral activation for diabetic retinopathy prevention (BADRP) • Education principles about diabetes mellitus • Behavioral therapy, and the health belief model to assist in identifying barriers to obtaining dilated fundus examination (DFEs) • Problem-solving solutions to surmounting barriers, • Action plans to facilitate DFEs	60 min per sessions/4 months	4 sessions	Individual/Community health worker	Pre-intervention, 6 months after intervention	At 6 months, BADRP participants were more likely to have a DFE than ST participants (85.7% vs. 51.1%; χ^2^ = 24.9, *p* < 0.001), with a risk difference of 0.538 (95% CI: 0.40–0.64; *p* < 0.001)
10	Crossland et al. [27]	General practitioners and practice nurses received training • In-house diabetic retinopathy screening implemented • Teleophthalmology support provided for mild to moderate cases without sight-threatening pathology • Quarterly videoconference education sessions for 12 months attended by GPs and ophthalmologists	​	4 main sessions	Individual/General practitioner and ophthalmologists	Pre-intervention, Within 3 years after intervention	Recorded screening rates were 100% across intervention practices, compared with 22%–53% in control practices
11	Zangalli et al. [28]	A multipronged intervention: Education-and telephone-based intervention • Educational brochure about diabetic eye disease: the importance of early diagnosis, the lack of symptoms in the early stages of the disease, and the effectiveness of early treatment in preventing blindness • A personalized letter reminder to schedule • An automated phone call prior to the scheduled visit	​	3 main activities	Individual	Within 3 months after intervention	Patients in the intervention group were significantly more likely to schedule (63% vs. 40%; *P* < 0.0001) and complete their appointment (48% vs. 30%; *P* < 0.0001) compared with usual care
12	Mwangi et al. [3]	Peer–led health education interventions • A monthly group health talk with structured content on diabetic eye disease and retinal screening as specified in the protocol • Individual monthly telephone reminders to attend eye exam	Monthly/90 days	2 main activities	Group, individual/Trained peer educators	During intervention	Eye exam uptake was higher in the intervention arm: of 104 participants, 31 (29.8%) attended screening—25/51 (49%) in the intervention group vs. 6/53 (11.3%) in the control group
13	Paudel et al. [29]	A community-based health education intervention • Community presentation on eye health: trained health workers in village health stations organised an educational community presentation on eye health • Loudspeaker broadcasting: once a day for a month to broadcast key health message in each village • Poster display: Health message posters about red eye, refractive error, cataract and posterior segment disease were displayed in commune health stations, schools and market malls • Brochure distribution: Provided to households and health stations with info on common eye diseases, symptoms, eye exam recommendations, and available eye care services	/1 month	4 main activities	Group of participants/Health workers	Baseline and 6 months after intervention	Intervention group showed significantly higher awareness and knowledge of eye diseases and red-eye prevention (OR 2.1–4.1, *p* = 0.03–0.0001) than controls
14	Na nakorn et al. [30]	A perceived benefit promotion program based on Pender’s health promotion model • Teaching: building relationships, raising awareness of eye health benefits, promoting exercise, healthy diet, proper eye drop and eyeglass use, regular eye exams, and sharing experiences • Demonstration and trained to practice: Demonstrate and practice appropriate exercises, eye exercise management, ask questions and share a record of actions to promote eye health • Discussion and telephone follow up: group discussion for exchange knowledge and experience • Prompting to action and home visit phone calls for providing support and encouragement to practice an eye health behavior	60 min per sessions/4 weeks	4 main activities	Groups of participants/Ophthalmic nurse specialty	Pre- intervention, Post- intervention	The mean score of eye health promoting behavior of older persons in the experimental group receiving perceived benefit promoting program was significantly higher than control group
15	Boonruan et al. [31]	Health education program using Pender’s health promotion model • Learning activities: focus groups, illustrations, handbooks, Q&A, and experience sharing • Demonstration/practice: eye exercises, facial massage, food selection, and Q&A • Reinforcement: Trigger Record form and reminder messages • Social support: emotional, eye massage media, manuals, and informational support via public health broadcasts	/8 weeks	4 main activities	Groups of participants/Ophthalmic nurse specialty	Pre- intervention, Post- intervention	The experimental group showed significantly higher perceived benefits, self-efficacy, and eye health promotion behaviors than the comparison group, while perceived barriers were significantly lower
16	Sapru et al. [32]	A mobile community-based eye health education program applying RE-AIM (reach, efficacy, adoption, implementation, maintenance) • The workshop: a glaucoma video followed by a discussion • Comprehensive eye exams: visual acuity, auto refraction, pupil examination, fundus photo, visual field, slit lam ophthalmologist’s examination at the onsites • The on-site treatment: laser procedures and/or eye drops • Provided follow-up services at 4–6 weeks and 4–6 months	60 minutes- long workshop	4 sessions	Groups of participants/Community health educators, eye technician, glaucoma specialist	Pre- intervention, Post- intervention	Knowledge scores increased significantly from pre- to post-test in both groups: intervention (3.86 → 4.97, p < 0.001, n = 707) and comparison (3.17 → 3.97, *p* < 0.001, n = 518)
17	Panchal and batra [4]	Structured teaching programme (STP) • Risk factors of visual impairment • Causes of visual impairment: cataract, glaucoma, age related macular degeneration, diabetic retinopathy • Sign and symptoms of visual impairment • Early detection and prevention of visual impairment	​	1 session	Group of participants	Pre- intervention, 7–10 days post intervention	Posttest scores were higher in the experimental group than the control group: knowledge (29.98 ± 2.81 vs. 17.88 ± 3.12, p < 0.001) and attitude (89.22 ± 5.96 vs. 74.74 ± 9.28, *p* < 0.001)
18	Ramagiri et al. [5]	Health education intervention with watched videos • The videos highlighted key information on diabetes and its effects on the eye, as well as the importance of annual screening, answered all queries and shared the address of local eye care facilities	​	1 session	Group	Within 2 months	Uptake of diabetic retinopathy screening was higher with the educational video than the pamphlet (32.7% vs. 11.5%; *p* < 0.05)
19	Müller et al. [33]	Eye health promotion campaign • Campaign messages aired via TV, radio, and newspapers • Three TV commercials screened and radio broadcasts targeted the audience throughout the day • Messages featured in two major metropolitan newspapers	​	3 main activities	Public campaign	12 months post intervention	The proportion of participants visiting an eye specialist in the past year rose from 61% to 70% (*p* < 0.001); those with diabetes receiving a dilated fundus exam in the past 2 years increased from 52% to 70% (*p* < 0.001); and consistent sunglasses use rose from 33% to 39% (*p* < 0.001)
20	Hark et al. [28]	A community-based intervention • Community workshops (45–60 min) and flyers/posters to raise glaucoma awareness and perform focused ocular exams • Community-based management, treatment, follow-up, and referral with telephone reminders for appointments	​	2 sessions	Individual, Group/Community health educators, ophthalmologist, ophthalmic technician	1 week after workshops	A total of 1,056 individuals attended the glaucoma detection examination after the education workshops and promotional materials
21	Umaefulamet al. [34]	Mobile health intervention via text message • The dissemination of diabetes- eye related text messages to participants for 12 weeks directly via mobile phone SMS	1- Daily message/12 weeks	​	Individual	Post- intervention	The DR knowledge, attitude, and practice scores significantly improved. Individuals living with diabetes had increased DR attitude and practice post-scores compared to those at risk of diabetes
22	Rhodes et al. [35]	Eye health education intervention • Patient videos: Two short iPad videos emphasizing the importance of routine dilated comprehensive eye exams (CEE) for those at risk of glaucoma, shown while pupils dilated • Brochures: Colorful, concise inserts highlighting glaucoma and the need for routine CEE • Posters: One poster at the Vision center, Walmart optical shop, and Walmart Pharmacy, with three posters per study site	3 min long per video	3 main sessions	Group of participants	2–4 weeks post- intervention	Patient knowledge and attitudes improved significantly (*p* ≤ 0.01). While 63% had a CEE in the past 2 years, 98% reported likely to have one in the next 2 years

RR: relative risk; CI: confidence interval.

### Assessment of Bias

**FIGURE 2:**
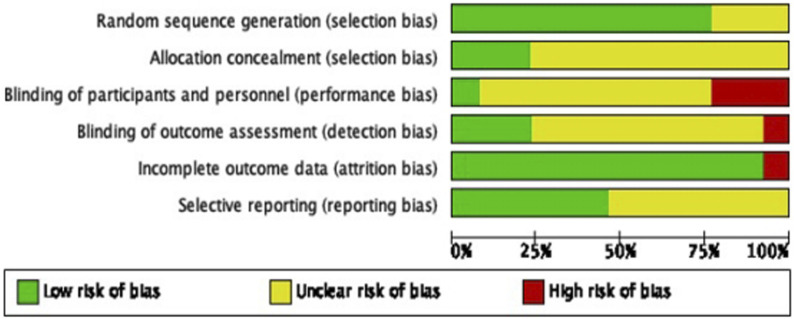
Critical appraisal of eligible randomized controlled trial studies (Thailand, 2025).

Thirteen studies were evaluated for bias risk. Most had low risk for random sequence generation (76.92%) and three studies (23.08%) had low risk for allocation concealment. However, blinding of participants (23.08%) and blinding outcome assessment (23.08%) had higher risks. Detailed information on the use of blinded research assistants was often lacking. Most studies had low risk for incomplete outcome data (92.30%) and selective reporting (46.15%). Seven studies (53.85%) were unclear due to unidentified protocols or trial registrations for cross-checking (Figure 2).

**FIGURE 3:**
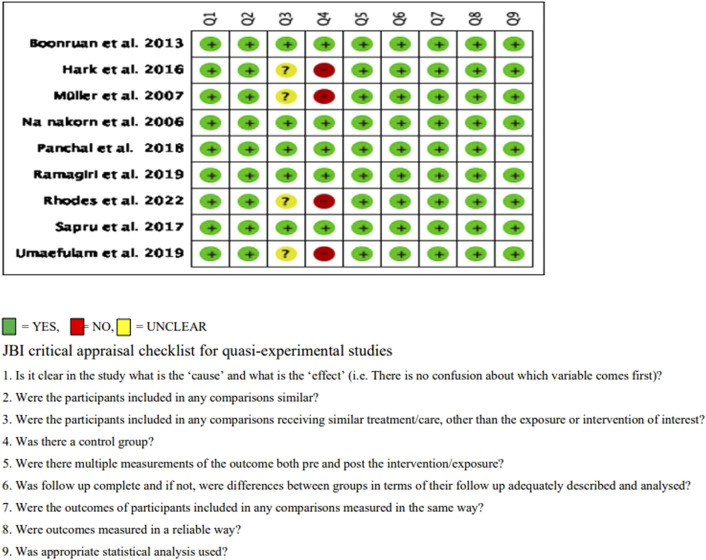
Critical appraisal of eligible controlled clinical trial and quasi-experimental studies (Thailand, 2025).

**FIGURE 4:**
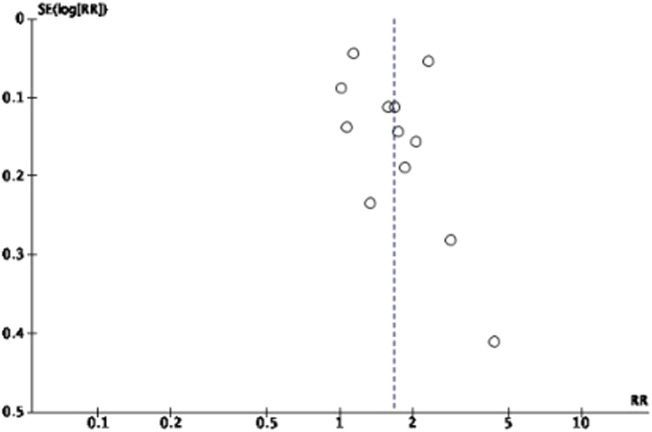
The publication bias of eye examination rate outcomes (Thailand, 2025).

Nine included studies were assessed for risk of bias using standardized critical appraisal instruments from the Joanna Briggs Institute Meta-Analysis of Statistics Assessment and Review Instrument (JBI-MAStARI). The criteria scored for quasi-experimental studies were cut, resulting in five studies receiving a 9/9 score with higher quality papers. The remaining studies were found to have a 7/9 score (Figure 3). The overall quality of evidence (GRADE) was rated at moderate for eye examination rate, and low for eye health attitude, eye health knowledge, and eye health behaviors. Lack of blinding, allocation concealment, and small sample sizes were reasons for downgrading the overall quality scores (Supplementary Appendices SB). Funnel plots were used to assess publication bias for eye examination outcomes (Figure 4). Other outcomes were not analyzed due to the limited number of studies and insufficient power to detect asymmetry.

### Effectiveness of Community-Based Interventions on Outcomes

#### Eye Health Attitude

**FIGURE 5:**
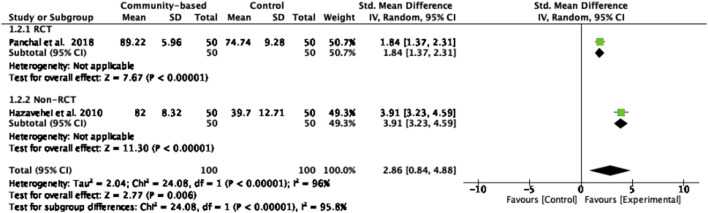
Forest plot of the effect of community-based interventions on eye health attitude (Thailand, 2025).

Two studies [4, 22], comprising 200 participants, reported eye health attitude. They were included in a meta-analysis using a random-effect model. The pooled analysis revealed that intervention groups exhibited significantly improved eye health attitude compared to control groups (z = 2.77, *p* = 0.006), with a large effect size (SMD = 2.86). The educational program [22] demonstrated a larger effect size (SMD = 3.91) compared to the structured teaching program [4] with an effect size of SMD = 1.84. Substantial heterogeneity in effect sizes was observed across the studies (*I* ^*2*^ = 96%, *Chi* ^*2*^ = 24.08, *p* < 0.00001) (Figure 5).

#### Eye Health Knowledge

**FIGURE 6:**
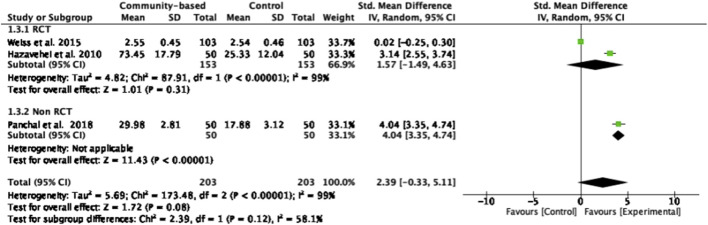
Forest plot of the effect of community-based interventions on eye health knowledge (Thailand, 2025).

Three studies [4, 22, 26], comprising 406 participants, reported eye health knowledge as an outcome. They were included in a meta-analysis. The overall effect of community-based interventions on eye health knowledge was not statistically significant (z = 1.72, *p* = 0.08), the range of effect sizes was substantial, varying from 0.02 to 4.04. Notably, the structured teaching program [4] demonstrated the largest effect size (SMD = 4.04). However, heterogeneity tests indicated significant variability across the three studies (*I* ^*2*^ = 99%, *Chi* ^*2*^ = 173.48, *p* < 0.00001) (Figure 6).

#### Eye Examination

**FIGURE 7:**
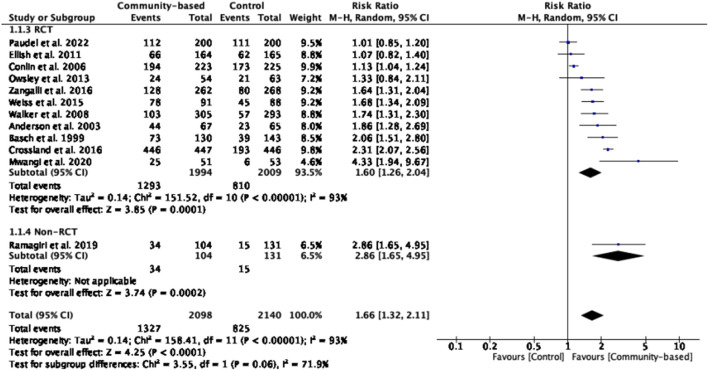
Effect of community-based intervention on eye examination rate (Thailand, 2025).

Twelve studies, encompassing 2,152 participants, reported eye examination rate and they were included in the meta-analysis [3, 5, 18–21, 23, 24, 26–29]. The effect of community-based interventions in enhancing overall eye examination among intervention groups was statistically significant (z = 4.23, *p* < 0.0001) with risk ratios ranging from 1.01 to 4.33. Notably, older people receiving community-based support groups (DSGs) with a peer-led health education had higher eye examination rate than those in the control group [3]. However, high heterogeneity was observed across these studies (*I* ^*2*^ = 93%, *Chi* ^*2*^ = 157.77, *p* < 0.00001) (Figure 7).

#### Visual Acuity

In a RCT [25], participants at risk for developing choroidal neovascularization received a home monitoring device as part of the community-based intervention. Results showed a smaller decline in visual acuity, with fewer letters lost compared to standard care (median, −4 letters; interquartile range [IQR], −11.0 to −1.0 letters), resulting in better visual acuity at choroidal neovascularization detection in the device arm.

#### General Eye Health Behavior

**FIGURE 8:**
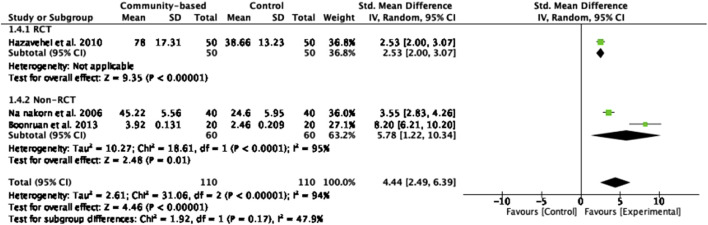
Forest plot of the effect of community-based intervention on eye health behavior (Thailand, 2025).

Three studies with 220 participants reported eye health behavior as an outcome [22, 30, 31]. Higher scores indicated better behavior. The effect of community-based interventions in improving EH behavior was statistically significant (z = 4.46, *p* < 0.00001), with a large effect size (SMD = 4.44). The effect sizes of three studies ranged from 2.53 to 8.20. In addition, a study implemented a health education program emphasizing on health promotion reported the highest effect size on eye health behavior among older persons in community [31]. However, the analysis reveals high heterogeneity among the studies (*I* ^*2*^ = 94%, *Chi* ^*2*^ = 31.06, *p* < 0.00001) (Figure 8).

#### Specific Eye Health Behavior

##### Intention Toward Eye Care Behaviors

A RCT [22] compared the effect of an educational program on intention toward eye care behavior to prevent diabetes ocular complication. The intervention group showed a statistically significant increase in scores related to eye care behavioral outcomes, attitude toward eye care behavior, enabling factors regarding preventing diabetic ocular complication, normative beliefs for employing methods to prevent blindness, subjective norms of participation in the process of eye care behavioral change, and intention toward eye care behavior compared to the control group immediately after and 3 months after the intervention.

##### Perceive Self-Efficacy to Perform Eye Health Behaviors

A quasi-experimental study [31] investigated the effectiveness of a health education program applying Pender’s Health Promotion Model for promoting eye health for the elderly. The result found that participants in the health education group exhibited significantly higher perceived self-efficacy to perform eye health promotion behaviors than the comparison group (*t* = 5.81, *p* < 0.001).

##### Sunglasses Usage Behavior

One randomized controlled trial [29] investigated the effectiveness of a community-based health education intervention in promoting sunglasses use among individuals aged over 60 years. Participants in the control were more likely to wear sunglass compared to those in control group. Odds ratios ranged from 2.1 to 4.1 (*p* = 0.03 to 0.0001).

##### Eye Care Behaviors Regarding Diabetic Retinopathy

In a quasi-experimental study [34] examined the changes in eye care behavior regarding diabetic retinopathy as a result of mobile health education intervention among Indigenous women with diabetes or at-risk of diabetes. The result found the individuals living with diabetes showed increased eye care behavior regarding diabetic retinopathy in the intervention group compared to those at risk in the control group.

##### Vision- Targeted Health-Related Quality of Life

In a randomized controlled trial [26], a diabetic retinopathy prevention program using behavioral activation showed a slight improvement in vision-targeted health-related quality of life in the intervention group compared to the supportive therapy group at the 6-month follow-up.

### Subgroup Analyses and Moderator Effects

Since four outcomes were assessed in the meta-analysis, subgroup analyses and moderator effect exploration were conducted only for eye examination uptake, as this outcome had a sufficient number of studies for meaningful statistical testing. Within this outcome, subgroup analyses were performed based on intervention characteristics, study characteristics, and sample characteristics. Subgroup analyses of eye examination outcome showed no significant differences between active and passive controls. Considerable variation was observed across measurement points, intervention formats, durations, single versus combined interventions, and intervention types, with high heterogeneity persisting in most subgroups (Table 3). Consistent with these findings, meta-regression analyses did not identify any statistically significant moderators of intervention effects, including publication year, study region, mean age, proportion of female participants, intervention type, duration, mode of delivery, and comparator (all p-values >0.05; see Table 4).

**TABLE 3:** Subgroup analysis of eye examination outcome (Thailand, 2025).

Subgroup	Number of studies	Sample size	Risk ratio (95% CI)	Z (p-value)	Heterogeneity		Subgroup different	
					Chi2 (*p*-value)	I2 (%)	Chi2 (*p*-value)	I2 (%)
1. Comparator								
Active control	7	1,647	1.65 (1.54,1.75)	15.25 (0.00001)	146.78 (0.00001)	96	0.57 (0.45)	0
Passive control	5	505	1.55 (1.35,1.78)	6.15 (0.00001)	13.40 (0.009)	70		
2. Measurement point of time								
<3-month	3	288	2.47 (1.35,4.54)	2.93 (0.003)	8.78 (0.01)	77	13.29 (0.004)	77.4
3–6-month	5	746	1.44 (1.08,1.92)	2.49 (0.01)	27.71 (0.0001)	86		
12-month	3	557	1.48 (1.03,2.14)	2.11 (0.03)	18.53 (0.0001)	89		
3-year	1	639	2.31 (2.07,2.56)	15.40 (0.00001)	-	-		
3. Intervention approach								
Individual	6	2,772	1.67 (1.20,2.33)	3.05 (0.002)	118.11 (0.00001)	96	3.35 (0.19)	40.3
Group	4	1,081	1.32 (0.93,1.87)	1.57 (0.12)	13.71 (0.003)	78		
Combine	2	377	2.72 (1.33,5.57)	2.74 (0.006)	2.96 (0.09)	66		
4. Intervention intensity								
<3-month	2	504	1.99 (0.45,8.75)	0.91 (0.36)	13.06 (0.0003)	92	0.76 (0.68)	0
3–6-month	3	1,106	1.47 (1.09,1.97)	2.54 (0.01)	7.94 (0.02)	75		
Others	7	2,620	1.77 (1.26,2.50)	3.25 (0.001)	129.17 (0.00001)	95		
5. Intervention type								
Combine	9	3,368	1.66 (1.27,2.18)	3.68 (0.0002)	84.84 (0.00001)	91	0.00 (0.98)	0
Single	3	862	1.65 (1.03,2.64)	2.08 (0.04)	25.32 (0.00001)	92		
6. Intervention type								
Letter reminder	1	67	1.86 (1.23,2.69)	3.26 (0.001)	-	-	0.35 (0.99)	0
Telephone call	1	123	1.74 (1.31,2.30)	3.85 (0.0001)	-	-		
Behavioral activation	1	123	1.68 (1.34,2.09)	4.59 (0.00001)	-	-		
Health education	7	1980	1.63 (1.20,2.21)	3.10 (0.002)	39.51 (0.00001)	85		
Tele-retinal imaging	2	1,341	1.61 (0.76,3.45)	1.24 (0.22)	120.96 (0.00001)	99		

**TABLE 4:** A moderator effect for an *eye examination* outcome (Thailand, 2025).

Moderators	Coefficient (β)	*p*-value	95% CI for β	R^2^ (%)	Residual I^2^ (%)
1. Study characteristic					
*-Regional (Reference: Africa)*	​	​	​	22	84.3
America	−1.055	0.079	[−2.262, 0.152]	​	​
Asia	−1.088	0.090	[−2.392, 0.216]	​	​
Australia	−0.63	0.316	[−1.988, 0.728]	​	​
2. Intervention characteristic					
*-Intervention Type (Reference: Individualized)*	​	​	​	0	84.6
Health education	0.399	0.385	[−0.739,1.537]	​	​
Behavioral activation	0.448	0.437	[−0.993,1.889]	​	​
Screening	0.767	0.207	[−0.647,2.181]	​	​
Teleretinal imaging	0.055	0.919	[−1.357,1.466]	​	​
Personalized follow up	0.550	0.367	[−0.953,2.053]	​	​
Supported group	1.397	0.100	[−0.419,3.213]	​	​
Telephone	0.483	0.411	[−0.979,1.945]	​	​
*-Intervention Mode (Reference: Combine)*	​	​	​	9.40	78.50
Single	0.134	0.634	[−2.499, 2.767]	​	​
*-Methodology (Reference: CCT)*	​	​	​	9.8	90.1
RCT	−0.951	0.218	[−1.591, 0.409]	​	​
*-Comparator (Reference: Passive control)*	​	​	​	0	91.50
Active control	−0.70	0.784	[−0.622, 0.483]	​	​
*-Measure time (Months)* *(Reference: >12)*	​	​	​	100	0
0–3	−0.208	0.244	[−1.911,1.496]	​	​
3–6	−0.823	0.786	[−2.355,0.709]	​	​
6–12	−0.671	0.251	[−2.216,0.874]	​	​
-Duration (Months) (Reference: 6)	​	​	​	43.4	8
1	−0.089	0.190	[−0.240,0.062]	​	​
3	0.530	0.137	[−0.239,1.298]	​	​
4	0.419	0.318	[−0.552,1.390]	​	​
3. Sample characteristic					
*-Age mean group (Years)* *(Reference: 70–79)*	​	​	​	0	91.40
<70	0.147	0.639	[−0.0.538, 0.832]	​	​

## Discussion

In this review, the methodological rigor of the systematic review and meta-analysis was carefully considered. The search strategy was comprehensive, covering multiple databases and additional sources, with predefined inclusion and exclusion criteria to minimize bias and ensure the relevance of the studies. Quantitative synthesis was conducted using appropriate meta-analytic techniques, including assessments of heterogeneity and sensitivity analyses, to ensure that the pooled estimates reliably reflected the underlying evidence. These methodological steps support the reliability and validity of the findings.

The findings from this systematic review and meta-analysis indicate that community-based interventions positively influence eye health attitudes, eye health knowledge, examination rates, eye health behaviors, and vision-related quality of life in older persons. However, the observed high heterogeneity across studies warrants careful consideration when interpreting the pooled effect sizes. Similarly, previous review reported that a variety of educational programmes resulted in improvements in knowledge about accessing eye care, improving attendance at screening improving attendance at follow-up eye exams among non-dominant group of people include older people [36].

Community-based interventions significantly improved attitude scores in intervention groups with large effect sizes (z = 2.77, *p* = 0.006, SMD = 2.86). Notably, educational programs [22] utilizing various forms of training including lecture, question and answer, group discussion, and practical presentation related to the knowledge of eye diseases, complications of eye diseases, appropriate food and exercise, the risk of eye diseases, monitoring, and visiting an ophthalmologist regularly to prevent ocular complications, resulted in increased patient attitudes and actions toward eye care. Structured teaching programs [4] improving the knowledge and attitude of older individuals about risk factors and causes of visual impairment, such as cataract, glaucoma, and diabetic retinopathy, were effective in enhancing their understanding of early detection and prevention of visual impairment, although statistical significance was not observed which may be attributed to several key factors related to the design and implementation of the interventions [26]. Firstly, variations in intervention components such as content, delivery methods (for example, lectures, pamphlets, and audiovisual aids), intervention duration, the measurement tools or scales used in a study and the expertise of facilitators can lead to inconsistent outcomes across studies. These differences may reduce the ability to detect meaningful effects, especially when the tools used to measure outcomes are not sensitive to small changes in knowledge and attitudes. Secondly, participant-related factors such as literacy levels, and pre-existing beliefs about eye health may influence how information is received, retained, and translated into preventive eye health behavior knowledge. Older persons may require tailored educational strategies that consider sensory and motivational needs to ensure effective engagement. Thirdly, short follow-up durations in many of the studies might not adequately capture the sustained impact of educational interventions. Future research should aim to standardize intervention components, use larger sample sizes, include control groups, and extend follow-up periods. It should also assess eye health–related knowledge, such as preventive behaviors, risk factors, and screening practices, alongside knowledge and attitude measures for a more comprehensive understanding of intervention impact.

Our review indicated older persons attending community-based interventions were more likely to take eye examination in comparison to controls (z = 4.23, *p* < 0.0001, RR = 1.01–4.33). Nonetheless, all five subgroup analyses did not achieve statistical significance except the one on measurement points. High heterogeneity was observed for almost all meta-analyses, which might result from various types of community-based interventions and small number of studies included in the subgroup analyses. However, the study implemented the community-based support groups with peer-led health education intervention including group health talk with content of eye diseases and telephone monthly reminders to attend the eye examination had the most effect on improving eye examination rate among older person in community [3]. Similarly, a previous review reported that recruitment initiatives and automated reminder calls were both highlighted as effective strategies to improve attendance and resulted in improvements in eye disease screening rates [36]. Studies with the following characteristics were associated with larger effect sizes on eye examination rates: (a) measuring short-term effects (less than 3 months post-intervention), (b) offering interventions in a combined individual and group format, and (c) delivering shorter intervention durations (less than 3 months). These findings suggest that community-based interventions can effectively improve eye examination rates through peer-led health education programs, which involve training community members to educate their peers about the importance of regular eye check-ups, share information on common eye conditions and available resources, and address misconceptions or concerns related to eye health. Peer-led approaches leverage trust and rapport within the community to promote behavior change toward proactive eye care. Although subgroup and meta-regression analyses did not identify statistically significant moderators, substantial heterogeneity persisted across studies. This may be due to limited variability in study and intervention characteristics, small sample sizes within subgroups, or unmeasured factors influencing intervention effects. These findings underscore the complexity of the interventions and suggest caution when interpreting pooled estimates. Future research with larger datasets and additional potential moderators is warranted to better understand sources of heterogeneity.

This study discovered that community health interventions significantly enhanced general eye health behavior, demonstrating a large effect size (z = 4.46, *p* < 0.00001), SMD = 4.44). Particularly, interventions lasting 1 month, featuring health education sessions through discussion and demonstration, active promotion of eye health practices, and utilizing emotional, material, and family support, proved to be the most effective [22, 30, 31]. The more the participants involved in the learning process, the better they retained the knowledge and felt confidence in eye health practice [30]. Moreover, the included study that utilized behavioral modification techniques was efficacious in enhancing eye health behavior [22]. Based on the results of the meta-analysis, it is possible to conclude that community-based interventions enhance general eye health behavior. These interventions achieve this by offering educational workshops and resources, conducting screenings for early detection of eye conditions, promoting healthy lifestyle habits such as regular eye examinations and proper eye protection, facilitating access to affordable eye care services, fostering community support networks, and raising awareness about the importance of eye health maintenance and prevention of blindness strategies. While the meta-analysis suggests that community-based interventions can improve general eye health behaviors, the heterogeneity of intervention outcomes highlights the influence of social and contextual factors. Variations in cultural beliefs, health literacy, socioeconomic status, and access to local health infrastructure may affect how interventions are received and implemented across different communities. Therefore, to maximize effectiveness, future programs should be context-specific, culturally sensitive, and adaptable to the unique needs of each target population. Tailoring content and delivery methods to local conditions may enhance engagement, sustainability, and the long-term impact of interventions on preventing visual impairment. Additionally, changes in health-related practices often require ongoing reinforcement to be maintained effectively.

Self-efficacy refers to an individual’s belief in their own ability to successfully perform specific tasks or behaviors to achieve desired outcomes [37]. Self-efficacy influences on motivation and effort. Greater dissatisfaction with substandard performance and stronger perceived self-efficacy for goal achievement resulted in a subsequent increase in effort intensity [37]. Regarding self-efficacy related to eye health promotion behavior, our review highlighted the effectiveness of health education interventions in individual study to enhancing perceived self-efficacy among older individuals. These interventions aimed to develop participants’ abilities and confidence in practicing eye health-promoting behaviors. This interventions improve self-efficacy related to eye health promotion behavior by offering educational programs, providing opportunities for eye health skill-building and practice, fostering supportive social networks, offering positive reinforcement and encouragement, and empowering individuals to take control of their eye health through achievable goal-setting [30, 31]. Integrating theoretical frameworks such as Pender’s Health Promotion Model, which was frequently used in the included studies, may enhance the understanding of how older adults engage in eye health practices. This model emphasizes perceived self-efficacy as a key determinant of health behavior. However, belief in one’s ability to take preventive action does not always lead to actual behavior change, especially among older persons who may face physical, cognitive, or environmental challenges. Therefore, future interventions should address both motivational factors and practical barriers to support sustained behavior change.

Visual acuity, as defined by Marsden, Stevens [6], is crucial for assessing the visual system’s function. It plays a vital role in screening for eye diseases like glaucoma, especially in early stages without apparent symptoms. Assessing visual acuity allows early detection, enabling timely intervention to prevent or manage eye diseases before they cause severe damage or symptoms. Chew, Clemons [25] found that daily testing with a home monitoring device significantly detected choroidal neovascularization progression. In summary, community-based interventions are essential for enhancing the screening process for eye diseases like glaucoma, particularly when symptoms are not apparent during the early stages. This is especially crucial for patients identified as being at high risk of developing rapidly progressing severe eye diseases. However, since this finding is based on a single study, the evidence remains limited, and further research is needed to confirm the effect of interventions on visual acuity outcomes.

Vision-related quality of life encompasses both physical and psychosocial aspects of vision. A holistic perspective including independence, emotional wellbeing, social interactions, and overall quality of life is crucial for understanding the effects of visual health on individuals’ wellbeing [38]. According to the review, community-based interventions that included behavioral activation through education on eye diseases, assistance in identifying barriers to obtaining eye care, problem-solving those barriers, and providing action plans for fundus examination significantly improved vision-targeted health-related quality of life [26]. A systematic review found that cataract surgery, anti-VEGF therapy for age-related macular degeneration, and treatment for macular edema enhanced quality of life compared to baseline or no intervention. [9]. Based on the analysis of relevant studies, community-based interventions, involving education on eye diseases, identifying and addressing barriers to eye care, and providing action plans for fundus examination, significantly enhanced vision-targeted health-related quality of life. However, since this finding comes from just one study, the evidence is still limited, and more research is needed to confirm how interventions affect vision-related quality of life.

In conclusion, our systematic review provides evidence supporting the positive impact of community-based interventions on various eye health outcomes among older persons. The implications for practice are evident, emphasizing the importance of tailored educational programs, health promotion activities, and structured interventions in enhancing eye health among older persons in community settings. However, based on the GRADE assessment, the evidence for improved eye health knowledge and positive eye health behaviors was low, suggesting that these outcomes can be generalized to similar community settings with caution. The evidence for enhanced eye examinations was low to moderate, indicating that this outcome may be more broadly applicable, while other aspects of eye examination had low-quality evidence, reflecting limited generalizability. It is important to recognise the study’s limitations, such as possible biases and differences in study designs, and approach it carefully when interpreting the findings. To enhance community-based interventions’ efficacy in promoting eye health in elderly populations, future studies should focus on specific aspects and strategies.

### Limitations and Future Research

Community-based interventions have shown potential in improving eye health outcomes among older persons. Due to the limited number of studies available for each outcome, particularly for eye health attitude, eye health knowledge, and eye health behaviors which were reported in only two to three studies, and the inconsistent reporting of key covariates, meta-regression was not conducted to further investigate potential moderators of intervention effects. These methods require a larger number of studies to produce reliable results. With such limited data, any attempt to explore the sources of heterogeneity would likely be unstable or misleading. This points to the need for more research to fill this gap and allow for a better understanding of what factors may influence the differences between study findings. Another limitation of this review is the inability to explore potential sources of heterogeneity through meta-regression or detailed subgroup analyses based on participant characteristics. Although factors such as age group, socioeconomic status, and baseline health conditions were considered as potential moderators, the primary studies included in the review lacked consistent and detailed reporting of these variables. This limited the extent to which population-level differences could be examined, and future studies should aim to report such characteristics more comprehensively. In addition, an important limitation of this review is the exclusion of several studies that initially met the inclusion criteria but were ultimately not included because their study populations were mixed (e.g., including younger adults or non-community-dwelling individuals) or because the interventions were implemented primarily in clinical settings rather than within community-based environments. As this review focused specifically on community-based interventions targeting older persons, studies conducted in hospitals or specialized clinics were excluded to maintain contextual relevance. However, this criterion may have reduced the number of eligible studies and limited the comprehensiveness of the review. Furthermore, many included studies recruited participants primarily from elder clubs or community groups, which may introduce selection bias. Members of such organizations often have greater health awareness and lead more active lifestyles compared to home-bound or institutionalized older persons. This limits the generalizability of the findings to the wider elderly population and underscores the need for future research to include more diverse and representative samples. Another limitation of this review is the inconsistency in the definitions and assessment methods of eye health outcomes across studies. While some studies evaluated clinical indicators such as visual acuity, others focused on behavioral or educational outcomes, including screening uptake, knowledge, and attitudes. This variability likely contributed to the high heterogeneity observed in the meta-analysis and posed challenges in synthesizing results. To address this, outcomes were categorized into relevant domains, and subgroup analyses were conducted when data permitted. Despite these efforts, the diversity in outcome measures and instruments limited comparability. Future research should prioritize the use of standardized and validated outcome measures to enhance consistency and facilitate more reliable evidence synthesis.

### Implications to Clinical Practice

Our findings suggested that community-based interventions could lead to positive changes in eye health attitudes, eye examination rates, and general eye health behaviors. Paticularly, structured teaching programs aiming at early detection and prevention of visual impairment can be offered to improve eye health knowledge and eye health attitudes. Educational programs utilizing various forms of training can be impletmented to increase patients’ knowledge and actions toward eye care, covering aspects such as eye disease knowledge, complications, healthy lifestyle practices, risk awareness, monitoring, and regular ophthalmologist visits to prevent ocular complications. To boost eye examination rate, peer-led health educations can be deliverd, which involve training community members to educate peers about check-ups, eye conditions, and resources. Short-term interventions with active promotion of eye health practices and social support are effective in fostering good eye health behaviors. Our findings highlight the potential of community-based strategies that are consistent with international frameworks such as the WHO’s Integrated People-Centred Eye Care (IPEC). These approaches can be adapted and tailored across diverse health systems, thereby reinforcing the relevance of our review beyond a single regional or national context. Therefore, governments and public health agencies should consider integrating this model into primary healthcare services, such as training community health volunteers, conducting community awareness campaigns, and organizing proactive eye screening units in remote areas, to improve access to prevention and treatment of eye diseases at early stages.

### Conclusions

Community-based interventions for older adults generally show a positive impact on eye health outcomes, including improved eye health attitudes, eye health knowledge, examination rates, eye health behaviors, and vision-related quality of life in older persons. However, the certainty of the evidence is low, with findings constrained by high risk of bias, substantial heterogeneity, and a small number of available studies, meaning results should be interpreted with caution. Based on the GRADE assessment, the evidence for improved eye health knowledge and positive eye health behaviors was low, suggesting that these outcomes can be generalized to similar community settings with caution. Evidence for enhanced eye examinations was low to moderate, indicating broader applicability, while other aspects of eye examination had low-quality evidence, reflecting limited generalizability. Overall, while these interventions are promising and suggest potential benefits, further rigorous, high-quality research is needed to confirm their effectiveness and determine the conditions under which they can be successfully implemented before they can be confidently recommended for widespread practice or policy adoption.
