# Supplementary material for: Community-Based Interventions to Improve Eye Health Outcomes in Older Adults: A Systematic Review and Meta-Analysis
Source: Public Health Rev. 2026 Jan 21;46:1607404. doi: 10.3389/phrs.2025.1607404 (PMC12867932; doi:10.3389/phrs.2025.1607404)
Supplement: Supplementary file 1 [file DataSheet2.pdf]

Appendices B: The overall quality of evidence (GRADE) (Thailand, 2025)

Author(s): Wasana Luangphituck[1], Plernpit Boonyamalik[2], Sunee Lagampan[3], and Chukiat Viwatwongkasem[4]
Question: Community-based intervention compared to control for improve eye health outcomes among older persons
Setting: Communities
Bibliography: [Community-based intervention] for improve eye health outcomes. Cochrane Database of Systematic Reviews [2024]

Certainty assessment							№ of patients		Effect		Certainty	Importance
№ of studies	Study design	Risk of bias	Inconsistency	Indirectness	Imprecision	Other considerations	Community-based intervention	control	Relative (95% CI)	Absolute (95% CI)		
**Eye health attitude - RCT**												
1	randomised trials	serious[a]	not serious	not serious	serious[b]	none	50	50	-	SMD **1.84 higher** (1.37 higher to 2.31 higher)	⊕⊕◯◯ Low	IMPORTANT
**Eye health attitude - Non-RCT**												
1	non-randomised studies	serious[a]	not serious	not serious	serious[b]	none	50	50	-	SMD **3.91 higher** (3.23 higher to 4.59 higher)	⊕⊕◯◯ Low	IMPORTANT
**Eye health knowledge - RCT**												
2	randomised trials	serious[a]	not serious	not serious	serious[b]	none	153	153	-	SMD **1.57 higher** (1.49 lower to 4.63 higher)	⊕⊕◯◯ Low	IMPORTANT
**Eye health knowledge - Non RCT**												
1	non-randomised studies	serious[a]	not serious	not serious	serious[b]	none	50	50	-	SMD **4.04 higher** (3.35 higher to 4.74 higher)	⊕⊕◯◯ Low	IMPORTANT
**Eye examination Comparator - RCT**												
11	randomised trials	serious[a,c]	not serious	not serious	not serious	none	1293/1994 (64.8%)	810/2009 (40.3%)	**RR 1.60** (1.26 to 2.04)	**242 more per 1,000** (from 105 more to 419 more)	⊕⊕⊕◯ Moderate	IMPORTANT

Certainty assessment							№ of patients		Effect		Certainty	Importance
**№ of studies**	**Study design**	**Risk of bias**	**Inconsistency**	**Indirectness**	**Imprecision**	**Other considerations**	**Community-based intervention**	**control**	**Relative (95% CI)**	**Absolute (95% CI)**		
**Eye examination Comparator - Non-RCT**												
1	non-randomised studies	serious[a]	not serious	not serious	serious[b]	none	34/104 (32.7%)	15/131 (11.5%)	**RR 2.86** (1.65 to 4.95)	**213 more per 1,000** (from 74 more to 452 more)	⨁⨁◯◯ Low	IMPORTANT
**Eye health promotion behavior - RCT**												
1	randomised trials	serious[a]	not serious	not serious	serious[b]	none	50	50	-	SMD **2.53 higher** (2 higher to 3.07 higher)	⨁⨁◯◯ Low	IMPORTANT
**Eye health promotion behavior - Non-RCT**												
2	non-randomised studies	serious[a]	not serious	not serious	serious[b]	none	60	60	-	SMD **5.78 higher** (1.22 higher to 10.34 higher)	⨁⨁◯◯ Low	IMPORTANT

CI: confidence interval; **RR:** risk ratio; **SMD:** standardized mean difference

Explanations

a. Lack of blinding

b. Small sample sizes

c. Lack of allocation concealment
