# Supplementary material for: Community-Based Interventions to Improve Eye Health Outcomes in Older Adults: A Systematic Review and Meta-Analysis
Source: Public Health Rev. 2026 Jan 21;46:1607404. doi: 10.3389/phrs.2025.1607404 (PMC12867932; doi:10.3389/phrs.2025.1607404)
Supplement: Supplementary file 2 [file DataSheet1.pdf]

Appendices A: Full search strategies (Thailand, 2025)

Publish database: PubMed
Search date: April 2023

PICO	Search terms	Record identified
Participants	1. older persons	445,592
	2. aging	628,404
	3. elderly	6,079,013
	4. “older adult”	13,697
	5. Senior	96,884
	6. 1 or 2 or 3 or 4 or 5	6,565,054
Interventions	7. community-based	86,428
	8. “community program”	637
	9. “community intervention”	1,791
	10. “primary care intervention”	268
	11. “eye health intervention”	49
	12. “eye health program”	37
	13. 7 or 8 or 9 or 10 or 11 or 12	90,249
Outcomes	14. eye	752,581
	15. vision	255,066
	16. “eye care”	8,172
	17. “eye health”	4,277
	18. “vision health”	361
	19. “ophthalmic care”	464
	20. “ocular care”	40
	21. “eye health attitude”	6,149
	22. “eye health knowledge”	13
	23. “eye examination rate”	11
	24. “visual acuity”	131,505
	25. “eye health behavior”	8,219
	26. “eye care practice”	34
	27. “vision-related quality of life”	1,137
	28. 14 or 15 or 16 or 17 or 18 or 19 or 20 or 21 or 22 or 23 or 24 or 25 or 26 or 27	928,883
	29. 6 and 13 and 28	7,742,824

Initial keywords will cover PICO framework: participants (older persons, aging, elderly, older adult, senior), intervention (community-based, community participation, community program, community intervention, primary care intervention), and outcomes (such as eye care, eye health, vision health, ophthalmic care, ocular care, eye health prevention, and eye health promotion).
Limit search: 1) Title Abstract and keywords
